# The role of inflammatory cytokines and ERK1/2 signaling in chronic prostatitis/chronic pelvic pain syndrome with related mental health disorders

**DOI:** 10.1038/srep28608

**Published:** 2016-06-23

**Authors:** Chao Hu, Hualan Yang, Yanfang Zhao, Xiang Chen, Yinying Dong, Long Li, Yehao Dong, Jiefeng Cui, Tongyu Zhu, Ping Zheng, Ching-Shwun Lin, Jican Dai

**Affiliations:** 1 Department of Urology, Ren Ji Hospital, School of Medicine, Shanghai Jiao Tong University, Shanghai, 200001, P. R. China; 2 Department of Urology, Zhongshan Hospital, Fudan University, Shanghai, 200032, P. R. China; 3 State Key Laboratory of Medical Neurobiology and Institute of Brain Science, Fudan University, Shanghai, 200032, P. R. China; 4 Liver Cancer Institute, Zhongshan Hospital, Fudan University & Key Laboratory of Carcinogenesis and Cancer Invasion, Ministry of Education, Shanghai, 200032, P. R. China; 5 Department of Urology, University of California, San Francisco, CA, 94143, USA

## Abstract

Mental health disorders(MHD) in chronic prostatitis/chronic pelvic pain syndrome (CP/CPPS) have been widely studied. However, the underlying role of inflammatory cytokines and their associated signaling pathways have not been investigated. Here, we report the potential role of cytokines and associated signaling pathways in CP/CPPS patients with MHD and in a CP/CPPS animal model. CP/CPPS patients (n = 810) and control subjects (n = 992) were enrolled in this case-control multicenter study, and serum cytokine levels were measured. Male Sprague-Dawley rats received multiple intracutaneous injections of an immuno-agent along with a pertussis-diphtheria-tetanus triple vaccine for autoimmune CP/CPPS development. The results revealed that, in CP/CPPS patients with significant MHD, elevated IL-1α, IL-1β, IL-4, IL-13, and TNF-α serum levels were observed. The above five cytokines in CP/CPPS rats were significantly elevated in prostate tissue (*p* < 0.05), and IL-1β levels were elevated in serum and cerebrospinal fluid. In behavioral tests, CP/CPPS rats showed anxiety- and depression-like symptoms, and impaired spatial and associative memory performance (*p* < 0.05). In the CP/CPPS group, ERK1/2 phosphorylation levels were increased in the amygdala and nucleus accumbens, and decreased in the hippocampus, but not caudate nucleus. Thus, prostate-derived cytokines, especially IL-1β, cross the blood brain barrier and may lead to enhanced ERK1/2 signaling in several brain areas, possibly underlying induction of CP/CPPS-related MHD.

Chronic prostatitis/chronic pelvic pain syndrome (CP/CPPS) is a commonly observed distress in male patients which is characterized by chronic pelvic discomfort, with voiding symptoms, sexual dysfunction, and associated mental health disorders (MHD)^1^. Though current antibiotic, alpha-blocker, and electro-magnetic therapy seems promising, the efficient treatment of CP/CPPS remains rare^2^,^3^,^4^. Therefore, there remains a high burden of MHD in CP/CPPS patients^5^,^6^. Among these MHD, depression has been identified in 78% of patients with CP/CPPS and 60% of them fall in the major depressive disorder category^7^. Male patients with CP/CPPS display higher stress levels and 62% of them show anxiety symptoms^8^,^9^. Hypothalamic-pituitary-adrenal axis dysfunction, pain, and personality changes have been suggested to be closely associated with the psychological profiles of CP/CPPS patients^6^,^8^,^10^. Farmer *et al*. reported specific pattern of functional brain activation and brain anatomical reorganization in CP/CPPS during neuroimaging studies, which hinted the potential correlation between peripheral dysfunction and central mechanism^11^. However, the investigation of inflammatory cytokines and their potential interactions with specific brain areas in CP/CPPS with MHD has rarely been reported. Increasing evidence support that autoimmunity is a major cause of dysfunction of the organs involved in CP/CPPS in both humans and in rodent models of autoimmune CP/CPPS^12^,^13^,^14^,^15^. Furthermore, inflammatory cytokines play a critical role in pathogenesis of autoimmune dysfunction and prove to be closely correlated to anxiety and depression^16^,^17^,^18^,^19^,^20^,^21^. Nevertheless, there is no study evaluating anxiety levels and depression-like behavior, and their correlation with inflammatory cytokine levels in human CP/CPPS or rodent models.

Over the last decades neuroscience research has identified specific brain areas, such as the basolateral amygdala (BLA), nucleus accumbens (NAc), hippocampus (Hippo), and caudate nucleus (Cau), which are related to anxiety, depression, and spatial and associative memory^22^,^23^,^24^,^25^. Synaptic plasticity is the cellular mechanism underlying cerebral physiological function^26^. Several reports have shown that the dysfunction of synaptic plasticity in the above brain areas is related to brain dysfunctions, such as anxiety, depression, and memory impairment^27^,^28^,^29^. The extracellular signal-regulated protein kinase (ERK1/2) signaling pathway is part of the complex network of signaling pathways implicated in synaptic plasticity, and the alteration of ERK1/2 signaling in neurons has been associated with impairment of structural plasticity, mood disorders, and MHD^30^,^31^.

Thus, to explore the molecular mechanisms underlying CP/CPPS associated MHD, psychological status and profiles of serum inflammatory cytokine levels were examined in CP/CPPS patients. Furthermore, behavioral performance of anxiety and depression, learning and memory disturbances, cytokine levels in prostates and cerebrospinal fluid (CSF), and alterations of ERK1/2 signaling were studied in specific brain areas in a rodent model of CP/CPPS.

## Results

### Elevated rate of anxiety and depression in CP/CPPS patients

Baseline clinical and mental parameters of recruited control subjects and CP/CPPS patients revealed no significant differences in age and BMI (Table 1). Significantly increased number of CP/CPPS patients displayed signs of anxiety or depression compared with control subjects as determined by higher scores with cut-off score of 50 (*p* < 0.05). Consistently, SAS, and SDS scores of CP/CPPS patients were significantly elevated compared with control subjects (*p* < 0.0001).

### Elevated serum cytokine levels in CP/CPPS patients and association with NIH-CPSI, SAS, and SDS

The serum levels of the cytokines IL-1α, IL-1β, IL-4, IL-6, IL-8, IL-10, IL-13, MCP-1, TNF-α and IFN-γ were measured in CP/CPPS patients and control subjects (Table 2). The levels of IL-1α, IL-1β, IL-4, IL-13, and TNF-α were significantly increased compared with control subjects (*p* < 0.05). Correlation analysis showed that the levels of IL-1β, IL-6, IL-8, and TNF-α of patients significantly and positively correlated with SAS and SDS (Table 2, *p* < 0.05). Interestingly, the levels of IL-10 and TNF-α were significantly and positively correlated with NIH-CPSI in patients with CP/CPPS (*p* < 0.05).

### Evaluation of the CP/CPPS rat model

#### Histological examination

**Figure 1:**
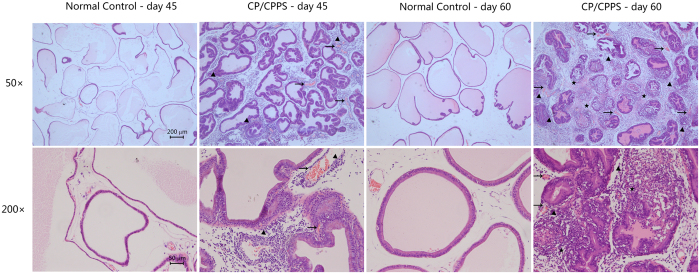
Hematoxylin and eosin stained sections of the prostates of controls and CP/CPPS rats. Normal control groups at day 45 and day 60, and CP/CPPS groups at day 45 and day 60 are shown. Inflammatory cell infiltration (▲) and vascular congestion (→) were observed at 50X magnification in the treatment groups. Ducts and glands damage (★) were visible at 200X magnification in the treatment groups. Scale bar is 200 μm for 50X magnification and 50 μm for 200X magnification.

After H&E staining of the prostates, N45 and N60 control groups showed normal prostatic histology with minimal inflammatory infiltration, duct changes, or tissue damage at 50x and 200x magnifications. However, the prostates of CP/CPPS rats showed a prominent increase in inflammatory infiltration, duct ectasia, and vascular congestion in the C45 group at both magnifications indicating that the autoimmune CP/CPPS model was successfully established in the individual rats. Furthermore, inflammatory duct infiltration and gland destruction was observed in the C60 group (Fig. 1).

#### Increased anxiety-like behavior in the CP/CPPS rat model

**Figure 2:**
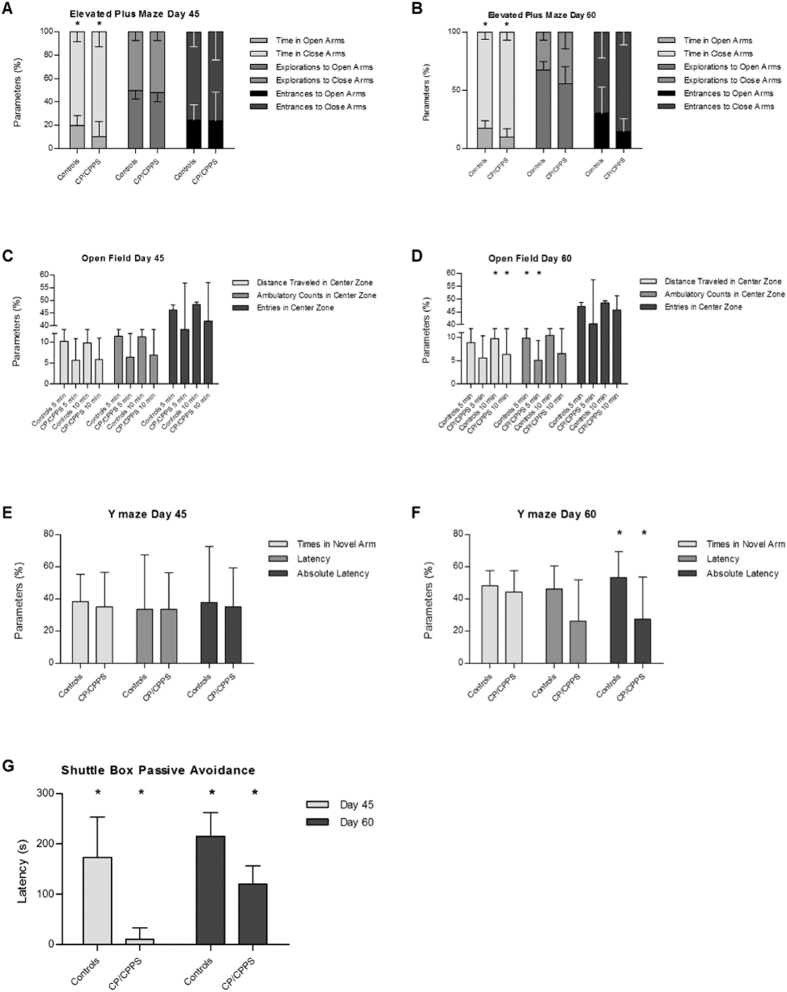
Behavioral analysis in controls (n = 8) and CP/CPPS rat model (n = 10). Elevated plus maze (EPM) performance ((**A**) day 45 and (**B**) day 60) of controls and CP/CPPS rats. Bars represent time (in %) spent in the open/closed arms, and explorations and entrances (in %) in the open/close arms in 5 min. Open field (OF) performance (**C**) day 45 and (**D**) day 60). Bars represent the distance traveled, ambulatory counts (total steps of rats in center zone), and entries into the center zone in the first 5 and 10 min. Y maze performance ((**E**) day 45 and (**F**) day 60). Bars represent time spent, latency, and absolute latency staying in the novel arm in 5 min during the 2^nd^ test. (**G**) Shuttle box passive avoidance (SBPA) performance at day 45 and day 60. Bars represent the latency in the light box on the 2^nd^ test day. ^*^Indicate a significance level of *p* < 0.05 versus controls. The data are presented as means ± SD.

In the EPM, the percentage of time spent in the open arms was significantly lower in the C45 group (10.42%) compared with the N45 group (Fig. 2A, 19.88%, *p* < 0.05). The percentage of time spent in the open arms was also lower in the C60 group (9.61%), when compared with the N60 group (Fig. 2B, 17.13%, *p* < 0.05). Furthermore, in both, day 45 and day 60 groups, the percentages of explorations of the open arms in the CP/CPPS groups were much lower compared with the control groups (day 45: 47.82% *vs*. 47.95%; day 60: 55.46% *vs*. 67.42%). Moreover, the percentages of entrances in the open arms in the C60 group (14.45%) was lower than that in the N60 group (30.33%), but no difference was observed between the C45 and N45 group (24.04% *vs*. 24.60%). Thus, the reduced activity in the open arms in the CP/CPPS groups reflect anxiety-like behavior.

#### Increased depression-like behavior in the CP/CPPS rat model

In both, the first 5 min and 10 min tests in the OF, the C45 group showed a lower percentage of distance traveled, ambulatory counts, and entries in the center zone compared with the N45 groups (Fig. 2C, 5 min: 5.67% *vs*. 10.15%, 6.43% *vs*. 11.39%, 37.08% *vs*. 46.13%; 10 min: 5.80% *vs*. 9.77%, 6.86% *vs*. 11.24%, 41.60% *vs*. 48.40%, respectively), but differences did not reach significance levels. During the first 5 min the percentage of ambulatory counts in the center zone in the C60 group (5.07%) was significantly lower compared with the N60 group (Fig. 2D, 9.82%, *p* < 0.05). Similarly, the distance traveled (5.59% *vs*. 8.87%) and number of entries (40.25% *vs*. 47.09%) into the center zone were lower, although these did not reach significance levels. During the entire test (10 min), the percentage of the distance traveled in the center zone in the C60 group (6.31%) was significantly lower compared to the N60 group (9.77%, *p* < 0.05). The percentage of ambulatory counts (6.51%) and entries (45.76%) in the center zone was lower compared to the N60 group (10.40% and 48.44%, respectively) without reaching significance levels. Therefore, the reduced activities in the center zone observed in the CP/CPPS groups compared to the control groups indicated depression-like behavior.

#### Impaired spatial memory in the CP/CPPS rat model

In the Y maze test, reduced time spent in the novel arm (34.96% *vs*. 38.21%) and the absolute latency (35.07% *vs*. 37.73%) was observed in the C45 group when compared with the N45 group, but the latency between the C45 and N45 group did not differ from each other (Fig. 2E, both 33.37%). In the novel arms of the Y maze, the percentage of the absolute latency in the C60 group (53.18%) was significantly shortened when compared with the N60 group (Fig. 2F, 27.36%, *p* < 0.05). Although the percentage of time spent in the novel arms (44.30%) and the latency (26.13%) in the C60 group were lower compared with the N60 group (48.12% and 46.12%, respectively), the differences were not statistically significant. Overall, the decreased activities at day 60 in the novel arms indicated that the CP/CPPS groups had difficulties in recollecting memory, which implies impaired spatial memory capacity in the CP/CPPS groups.

#### Impaired associative memory related behavior was observed in the CP/CPPS rat model

In the SBPA test (Fig. 2G), the latency in the C45 group was significantly lower compared with the N45 group (10.78 s *vs*. 172.87 s, *p* < 0.05). In addition, the latency in the C60 group remained significantly lower than that in the N60 group (120.28 s *vs*. 215.19 s, *p* < 0.05). Although rodents instinctively enter darkness, the previous foot shock provided a negative association with darkness. Thus, the lessened latency reflected impaired associative memory in the CP/CPPS groups.

#### The expression of cytokines and their receptors in prostate and specific brain areas associated with anxiety, depression, and memory

**Figure 3:**
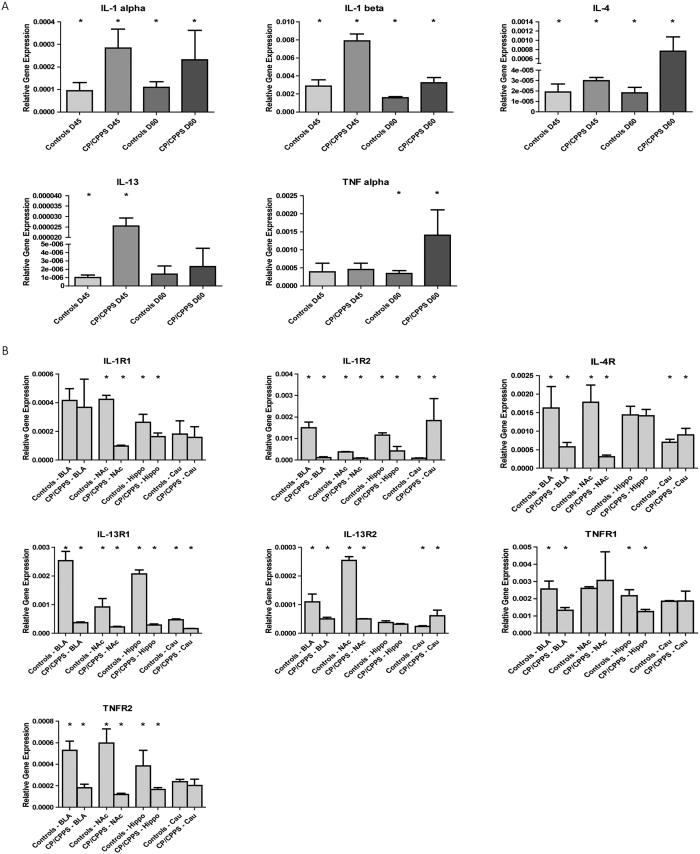
Quantification of the levels of IL-1α, IL-1β, IL-4, IL-13, TNF-α (**A**), and their receptors (**B**) in prostates of controls (n = 8) and CP/CPPS rats (n = 8) using RT-qPCR. Bars represent relative gene expression levels of cytokines and their receptors in prostates and brain regions (BLA, NAc, Hippo, and Cau) of control rats (D45 and D60) and CP/CPPS rats (D45 and D60). ^*^Indicate significance levels of *p* < 0.05 compared with controls. The data are presented as means ± SD.

In prostates, RT-qPCR showed that the levels of IL-1α, IL-1β and IL-4 in the CP/CPPS rats were significantly elevated compared with controls (*p* < 0.05, Fig. 3A). However, the difference in IL-13 between the N60 and C60 groups, and TNF-α between the N45 and C45 groups were not significant. Furthermore, in brain tissues the receptors of these cytokines, including IL-1R1, IL-1R2, IL-1R4, IL13R1, IL-13R2, TNFR1, and TNFR2, were detectable in BLA, NAc, Hippo, and Cau (Fig. 3B). Note that IL-13 and TNF-α both have two kinds of subtype receptors (IL-13R1 and IL-13R2, TNFR1 and TNFR2). The expression levels of the cytokine receptors IL-1R2, IL-4R, IL-13R1, IL-13R2, TNFR1, and TNFR2 were significantly reduced in BLA; IL-1R1, IL-1R2, IL-4R, IL-13R1, IL-13R2, and TNFR2 were significantly reduced in NAc; and IL-1R1, IL-1R2, IL-13R1, TNFR1, and TNFR2 were significantly reduced in Hippo. In contrast, IL-1R2, IL-4R, and IL-13R2 were significantly increased, and IL-13R1 was significantly reduced in Cau in CP/CPPS rats compared with controls.

#### Elevated cytokines in prostate correlated to MHD related behavioral pattern in rats

Correlation analysis using the Spearman rank correlation was performed for cytokines in the prostate and MHD related behavioral pattern. The significantly elevated IL-1α, IL-1β, IL-4, IL-13, and TNF-α levels in the prostate were all negatively related to positive responses in MHD related behavioral tests, which means positive correlation to anxiety, depression, and spatial and associative memory impairment, although significance levels were not reached (Table 3).

#### ERK1/2 activation in BLA and NAc and inactivation in Hippo and Cau in the CP/CPPS rat model

**Figure 4:**
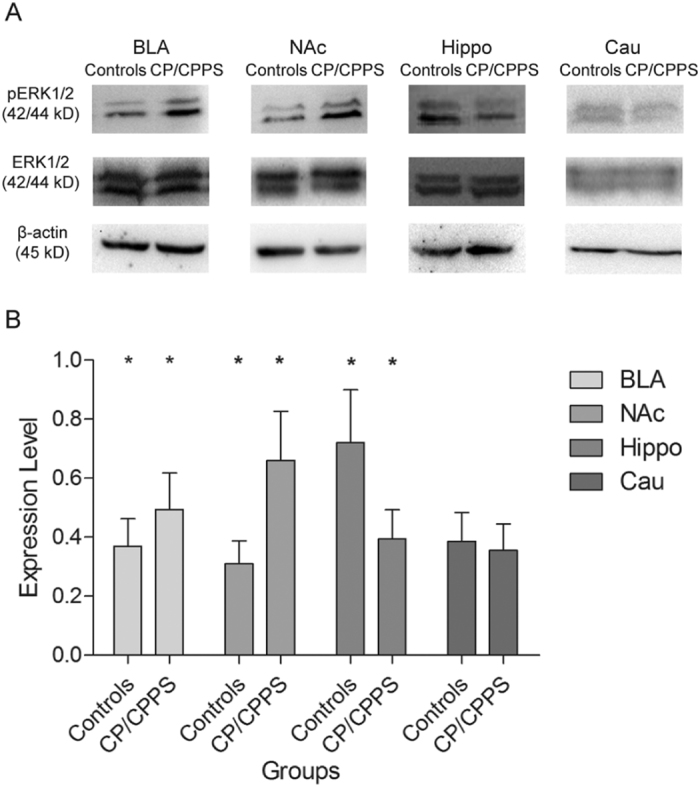
Western blot analysis of ERK1/2 activation in BLA, NAc, Hippo, and Cau in controls and CP/CPPS rats. (**A**) Representative Western blots. (**B**) Quantification of ERK1/2 phosphorylation level (pERK1/2) in BLA, NAc, Hippo, and Cau in controls (n = 8) and CP/CPPS rats (n = 8) using densitometric analysis of the bands shown in (**A**). Expression levels values normalized to β-actin levels. ^*^Indicate significance levels of *p* < 0.05 *vs.* controls. The data are presented as means ± SD.

Western blot analysis revealed that the phosphorylation level of the ERK1/2 was significantly increased in BLA and NAc of CP/CPPS rats compared with controls (*p* < 0.05), indicating enhanced ERK1/2 activation. In contrast, ERK1/2 phosphorylation levels were significantly reduced in the Hippo in CP/CPPS rats compared with controls (*p* < 0.05), indicating ERK1/2 inactivation in these brain regions. However, the phosphorylation level of ERK1/2 did not show alterations in Cau between CP/CPPS and control rats (Fig. 4A,B).

#### Elevated IL-1β in serum and CSF of the CP/CPPS rat models

**Figure 5:**
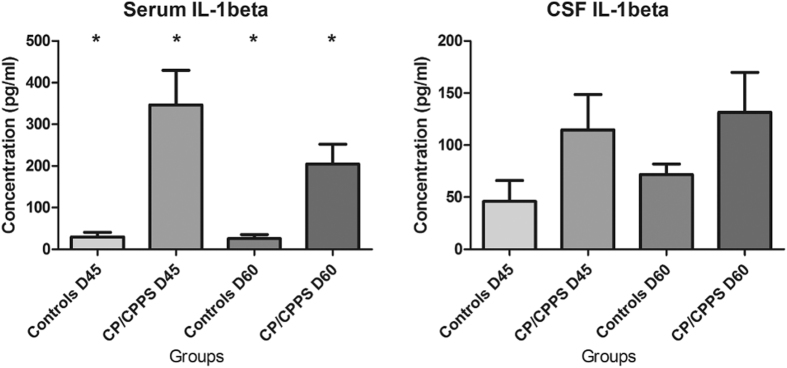
Quantification of the IL-1β in serum and CSF of controls and CP/CPPS rats. Bars represent the concentration (pg/ml) of serum and CSF IL-1β in controls (N45 and N60, n = 6) and CP/CPPS rats (D45 and D60, n = 7). ^*^Indicate *p* < 0.05 *vs.* controls. The data are presented as means ± SD.

In serum, the levels of IL-1β in both the C45 and C60 group were significantly increased compared with controls (*p* < 0.05). Similarly, in the CSF the level of IL-1β was elevated although the differences were not significant (Fig. 5).

## Discussion

The present study comprising 810 CP/CPPS patients with MHD and 992 control subjects showed significantly more anxiety and depression with higher SAS and SDS scores observed in CP/CPPS patients compared with normal control subjects, which was consistent with the previous reports^32^,^33^,^34^. Next, we investigated potential mechanisms that may facilitate CP/CPPS-related MHD. Five cytokines, IL-1α, IL-1β, IL-4, IL-13 and TNF-α, were significantly elevated in the serum of CP/CPPS patients. Among them, both IL-1β and TNF-α were positively correlated with SAS and SDS scores, which hint to the potential role of IL-1β and TNF-α in CP/CPPS-related MHD.

To further investigate the role of cytokines in CP/CPPS-related MHD, an established autoimmune rodent animal model of CP/CPPS was employed. Multiple intracutaneous injections at the neck, the tail, and the pelvic limbs with immuno-agent and an intraperitoneal injection with pertussis-diphtheria-tetanus triple vaccine for 45 and 60 days generated CP/CPPS in rats. CP/CPPS-related MHD in rats was evaluated by tests for anxiety in EPM, depression-related activity levels in OF, spatial memory impairment in the Y maze, and associative memory impairment in SBPA^35^,^36^,^37^,^38^. Similar to the finding in human subjects, anxiety-like and depression-like behavior was more common in the CP/CPPS rat model compared with control animals. Moreover, significant spatial and associative memory impairment was observed in the CP/CPPS group, what supports a possible link between disturbances in anxiety- and depression-like behavior and memory.

Similar to the findings in CP/CPPS patients, elevated levels of IL-1α, IL-1β, IL-4, IL-13, and TNF-α were observed in CP/CPPS rat prostates, and these elevated cytokine levels showed a positive trend in their correlation with anxiety, depression, and memory impairment. Even though significant levels were not achieved, there was a consistent trend for a correlation between elevated cytokine patterns and the behavioral performance of MHD, which suggested a potential valuable link between cytokines and CP/CPPS-related MHD. In the present study, we found that not only cytokine expression showed significant changes in specific brain areas, but that also the receptors of IL-1α, IL-1β, IL-4, and IL-13 showed decreased expression profiles, some of them reaching statistical significance levels, in BLA, NAc, and Hippo in CP/CPPS rats compared with controls. Interestingly, the changes of these receptors in Cau between CP/CPPS rats and controls seemed not predictable from the changes observed for their ligands. Thus, it is likely that rather than one kind of cytokine probably the integration of multiple cytokines and their receptors is important for the behavioral impact. Previous studies have demonstrated that cytokines by interacting with their specific receptors may modulate the MAPK/ERK downstream signaling pathway^39^,^40^. Through stabilizing structural changes in dendritic spines, MAPK/ERK regulates a wide variety of forms of synaptic plasticity. Synaptic plasticity is a crucial cellular mechanism underlying a variety of brain functions, including learning and memory, but also depressive and anxiety behavior. Interestingly, in anxiety and depression patients, ERK1/2 has been found activated in BLA and NAc^41^,^42^,^43^,^44^. However, during impairment of memory functions ERK1/2 has been shown to be inactivated in the Hippo and Cau^45^,^46^. Briefly, when ERK1/2 was inhibited synapse formation was reduced leading to the inhibition of synaptic plasticity^26^. Consistently, by using the 3T morphometric system, Mordasini *et al*. described reduction in relative gray matter volume in the anterior cingulate cortex, a core structure of emotional pain processing, correlating with bother of CP/CPPS. Even though the disaccord on brain area alterations were found compared to the demonstration of Farmer *et al*., both of these research units discovered the specific alteration in brain areas, which supported by the theory that dysfunctional central plasticity and functional reorganization lead to peripheral discomfort, such as chronic pain^5^,^11^. The functional magnetic resonance imaging and identification of different phases of CP/CPPS would probably be helpful in solving current divergence. In the present study, ERK1/2 phosphorylation levels were increased in the BLA and NAc and were reduced in the Hippo of CP/CPPS rats. The changes of this signaling pathway downstream of cytokines were consistent with the observed changes in behavioral test. We also noted that CP/CPPS rats showing reduced associative memory behavioral performance compared with controls, did not show changes in ERK1/2 activity. Therefore, these significant behavioral differences must be correlated with several other factors, besides ERK1/2, such as glucocorticoids and dopamine-dependent memory retrieval signaling pathways, which likely contribute to the associative memory impairment.

Cytokine levels showed similarly elevated trend in humans and in the CP/CPPS rat models, hence the PPE combined FCA induced autoimmune CP/CPPS appears to mimic the inflammatory status of CP/CPPS patients. Importantly, Leonard *et al*. have suggested that IL-1β could be transported from peripheral blood into the brain by an active transport mechanism^47^. Our results showed elevated IL-1β in CSF of the CP/CPPS rat model. It appears likely that these elevated CSF cytokines interact through some of the identified receptors for these cytokines in specific brain areas. Therefore, IL-1β derived from prostate may pass through the blood brain barrier, be transported by CSF, and interact with its receptors in specific brain areas.

Previous reports have shown that IL-1β may lead to anxiety by interacting with the endocannabinoid system and modulate neuronal function of the BLA^48^,^49^. Through participating in hypothalamic-pituitary-adrenal axis hyperactivity, IL-1β has been shown to cause depression^50^,^51^. In addition, IL-1β has been closely correlated with spatial and associative memory injury in previous studies^52^,^53^. In our study, IL-1β was significantly elevated in the CP/CPPS group and was positively correlated with SAS and SDS. Thus, the cytokine IL-1β may be one of the major molecular factors responsible for CP/CPPS-related MHD. Altogether, results in an animal model and in CP/CPPS patients confirm that anxiety and depression are associated with CP/CPPS. This study also confirms that elevated peripheral cytokine levels in the prostate and serum are likely involved in the molecular pathophysiology of CP/CPPS-related MHD. Taken together, it is a possibility that the prostate-derived cytokines, especially IL-1β, cross the blood brain barrier and can interact with their respective receptors in specific brain areas, which may lead to changes in ERK1/2 activity, and in turn lead to CP/CPPS related anxiety, depression, and spatial and associative memory impairment.

The present study has several limitations. The lack of significant differences in the activity of ERK1/2 made it difficult to assign a specific mechanism of action for prostate-derived cytokines in CP/CPPS-related MHD. The downstream ERK1/2 signaling pathway in neurons is conditioned by various kinds of factors, such cytokines interaction, growth factors, and ligands of G protein coupled receptors. The current results are probably an account of the balance of these effects. Further studies on the mechanism of action, such as perfusion with ILs, polyamines, and their antagonists by stereotaxic techniques and by *in vitro* chronic stimulation in primary cultures of specific brain areas are needed to address those issues. However, CP/CPPS-related MHD in patients as well as in rats and changes of peripheral inflammatory cytokines and downstream cerebral ERK1/2 signaling activity observed in the present study will undoubtedly give additional opportunity to explore uro-neurology diseases in this direction. Clinically, possibly blockage or depletion of inflammation cytokines in peripheral blood and CSF would be helpful not only in reducing inflammation, but also in developing available strategy in relieving CP/CPPS related MHD.

## Methods

### Ethics, consent and permissions

The clinical protocol of this study was reviewed and approved (No. 0015004) by the Institutional Ethical Review Committee, Ren Ji Hospital, School of Medicine, Shanghai Jiao Tong University, and after fully explaining the procedure and the aims of the investigation, all study subjects signed an informed consent form. The clinical trial was strictly executed in accordance with the approved guidelines and requirements. The rodent animal study was designed and conducted (No. SYXK 2013–0050) in accordance with the approval of the Animal Care and Use Committee of the School of Medicine, Shanghai Jiao Tong University.

### Study patients and design

Between July 2012 and August 2013, 1000 patients at seven hospitals (Ren Ji Hospital and Shanghai General Hospital affiliated with the Shanghai Jiao Tong University, Zhongshan Hospital affiliated with the Fudan University, East Hospital affiliated with the Tongji University, Shanghai Eighth Hospital affiliated with the Jiangsu Hospital, and Longhua and Yueyang Hospital affiliated with the Shanghai University of Traditional Chinese Medicine) who complained of discomfort or pain in the pelvic region (penis, testes, scrotum, perineum, pubic area, or lower back) for at least three of six months, were interviewed and examined by experienced urologists. Eight hundred and ten patients (aged 18–59), who were diagnosed with CP/CPPS based on the National Institute of Health (NIH) criteria, were enrolled in this case-control study^1^. Age matched healthy men (n = 1000) without any discomfort in the pelvic region were interviewed and examined by the urologists, and 992 were recruited as healthy controls.

All patients and healthy controls underwent physical and rectal examinations, hemanalysis, midstream urine analysis for microbe growth, and quantification of the prostate specific antigen (PSA). All patients and control subjects completed the Chinese versions of the NIH Chronic Prostatitis Symptom Score (NIH-CPSI), the Self Anxiety Scale (SAS) and the Self Depression Scale (SDS)^54^,^55^. Both SAS and SDS set scores of 50 as cut-off value with higher values being considered diagnostic for anxiety or depression. Within both groups, the serum samples of 56 individuals (28 patients and 28 controls) were randomly selected for analysis of inflammatory cytokine levels.

### Quantification of cytokine levels in the study subjects

The serum cytokine levels were assayed using a Quantibody Human Inflammatory Array (RayBiotech, Atlanta, GA, USA), which quantified the levels of interleukin-1α (IL-1α), IL-1β, IL-4, IL-6, IL-8/CXCL8, IL-10, IL-13, monocyte chemotactic protein (MCP)-1/CCL2, tumor necrosis factor (TNF)-α and interferon (IFN)-γ in the serum of the study subjects. The test was performed by multiplex sandwich enzyme-linked immunosorbent assay (ELISA) according to the manufacturer’s instructions. Assay sensitivity for all proteins tested was <1 pg/ml.

### Protein extraction from prostate tissue in rats

Four-month-old male Sprague-Dawley (SD) rats (n = 7, specific pathogen-free) received anesthesia with sodium pentobarbital (30 mg/kg, i.p.) under aseptic conditions. A lower abdominal incision was made and the prostate was identified and extracted. For protein analysis, the prostate was homogenized manually with 0.5% Triton X-100 lysis buffer and centrifuged (13,500 rpm, 30 min) twice at 4 °C. The supernatant was then collected as prostate protein extract (PPE). The PPE concentration was quantified and normalized to 40 mg/ml by spectrophotometry (NanoDrop ND-100, Wilmington, DE, USA).

### Establishment of CP/CPPS rat model

To establish a CP/CPPS animal model, two-month-old male SD rats were randomly divided into four groups, two CP/CPPS groups (C45 and C60) and two normal control groups (N45 and N60). The C45 (n = 10) and C60 (n = 10) groups were treated by the autoimmune method for 45 days or 60 days, respectively, to induce development of CP/CPPS. Briefly, the animals underwent multiple intracutaneous injections at the neck (0.4 ml), the end of the tail (0.3 ml), and the pelvic limbs (0.15 ml) with immuno-agent (1:1 mixed PPE and Freud’s complete adjuvant) and an intraperitoneal injection with pertussis-diphtheria-tetanus triple vaccine (0.5 ml). The corresponding normal control groups N45 (n = 8) and N60 (n = 8) received the same injection regimen for 45 and 60 days, respectively, but using saline. The autoimmune method was conducted at the 1st day and repeated at the 30th day.

### Histological examination

The inflammation status of the prostate was evaluated by visual examination of gland integrity, white blood cell infiltration, and structure of prostatic ducts through hematoxylin and eosin (H&E) staining. Briefly, the prostates from each group were immersed in 10% neutral buffered formalin, embedded in paraffin, and cut into 5 μm sections. Slides were cleared in xylene and dehydrated, followed by standard H&E staining procedure.

### Behavioral analysis of anxiety, depression, and memory

#### Elevated plus maze (EPM)

The potential effects of CP/CPPS in rats on anxiety-like behavior were assessed in the EPM. At the start of each trial, a single rat was placed in the open central square formed by the arms, facing the open arm. Time spent in open and closed arms, explorations, and entries to these arms were recorded and analyzed over a 5 min observation period by video camera system and analysis software (Med-Associates, St. Albans, VT, USA). The arms were cleaned thoroughly with 95% ethanol and water after each animal had been tested to prevent odor recognition. Lower activity in the open arms of the EPM, were suggestive of an anxiety-like condition^56^.

#### Open field (OF)

The potential effects of CP/CPPS in rats on depression-like behavior were assessed in the OF paradigm. A single rat was placed in the center of the OF apparatus. The distance traveled, ambulatory (walking) counts, and entries into the center zone of the OF were recorded and analyzed for a period of 10 min with a video camera system and analysis software (Med-Associates, St. Albans, VT, USA). The apparatus was cleaned thoroughly with 95% ethanol and water after each test session. Low locomotor activity levels in the center zone were considered depressive behavioral condition^57^.

#### Y maze

The potential effects of CP/CPPS in rats on spatial memory were assessed in the Y maze. On the first trial, rats were habituated individually to a randomly selected “start arm” for 5 min, one of the two remaining arms was randomly blocked as “novel arm”, whereas on the second trial, lasting for 5 min, all the arms of the maze were open. The two trials were separated by a 2 min break, during which the rat was returned to its home cage. Travel times into the novel arms, latency (*i.e.*, percentage of time in novel arms compared to total time), and absolute latency (*i.e.*, percentage of time in novel arms compared to total time without the time staying in the center zone) before moving in the novel arms were measured with a video camera system and analysis software (ANY-maze, Wood Dale, IL, USA). Between trials, the maze was cleaned using 95% ethanol and water and then dried. It was assumed that spatial memory was impaired when the activity in the novel arms was reduced^58^.

#### Shuttle box passive avoidance (SBPA)

The potential effects of CP/CPPS in rats on associative memory related behavior were assessed in the shuttle box (Med-Associates, St. Albans, VT, USA). During the training session, rats were accustomed to the behavioral apparatus in the light box and were free to move into the dark box. Afterwards, the rats were given a foot shock (1 mA, 2 s) whenever they entered the dark box. During the test session, the rats were placed in the light box again and no electric foot shock was applied. The session ended after the animal stayed in the light box for more than 300 s. All animals were tested for associative memory retention 24 h after the training session. The latency change between training and test session was recorded and the apparatus was cleaned thoroughly with 95% ethanol and water after each session. Previous reports have shown that associative memory was impaired when the latency in the light box on the 2^nd^ day was decreased^59^.

#### CSF and brain tissue collection

CSF of each rat was collected by fine needle puncture at the foramen magnum. Under anesthesia (sodium pentobarbital, 30 mg/kg, i.p.), rats were kept in a lateral position to allow the back of the neck to be fully exposed. The puncture was made at the level of the foramen magnum and CSF was collected and stored at −80 °C. After euthanasia, the rat brain was excised and the BLA, NAc, Hippo, and Cau were dissected according to The Rat Brain instruction and stored at −80 °C^60^.

#### Reverse transcription quantitative real-time PCR (RT-qPCR)

Cytokines IL-1α, IL-1β, IL-4, IL-13, and TNF-α mRNA expression levels were studied in the prostate, and the mRNA expression levels of the corresponding cytokine receptors, IL-1R1, IL-1R2, IL-4R, IL-13R1 (IL-13Rα1), IL-13R2 (IL-13Rα2), TNFR1 (TNFRsf1a), and TNFR2 (TNFRsf1b) were studied in the brain nuclei. Briefly, total RNA was extracted from TRIzol homogenates and purified using trichloromethane and isopropanol. cDNA was synthesized using RevertAid First Strand cDNA Synthesis Kit (Fermentas, Waltham, MA, USA). qPCR for cytokines was performed with Platinum^®^ RTS SYBR^®^ Green qPCR SuperMix-UDG (Invitrogen, Carlsbad, CA, USA) using specific primer sequences (Table 4). Briefly, initial activation was at 95 °C for 2 min, denaturation at 95 °C for 15 s, annealing at 60 °C for 30 s, and extension at 72 °C for 30 s with a single fluorescence measurement up to 40 cycles. A melting step with temperature ramps was set from 60 °C to 95 °C. The cycle time values were normalized against the endogenous control GAPDH.

#### Western blot analysis

Brain tissue from CP/CPPS rats was homogenized twice at high speed for 15 s in slurry tubes with magnetic beads in 1 ml ice-cold homogenization buffer. The extraction buffer consisted of radioimmunoprecipitation assay (RIPA) lysis buffer (200 ml), supplemented with protease inhibitor cocktail (P8340, 2 ml, 1%), phenylmethanesulfonyl fluoride (PMSF, 4 ml, 2%), Triton X-100 (1 ml, 0.5%), and protease inhibitor cocktail (10 ml, 5%; all Sigma-Aldrich, St. Louis, MO, USA), and quantified using the BCA method. After quantification, proteins (100 mg/lane) were subjected to 10–12% sodium dodecyl sulfate polyacrylamide gel electrophoresis (SDS-PAGE) to separate the proteins, and then electrotransferred onto polyvinylidene fluoride (PVDF) membrane. The membrane was incubated for 1 h at room temperature with blocking buffer (5% milk in TBS), and then further incubated with the relevant primary antibodies (phospho-MAPK (Erk1/2) mAb, Cell Signaling Technology, Beverly, MA, USA; MAPK (Erk1/2) mAb, Cell Signaling Technology, Beverly, MA, USA; and b-actin mAb, Santa Cruz Biotechnology, Santa Cruz, CA, USA) overnight at 4 °C. All primary antibodies were used at dilutions recommended by the manufacturer. After washing, membranes were incubated with the horseradish peroxidase (HRP)-conjugated secondary antibody (anti-rabbit IgG, Santa Cruz Biotechnology, Santa Cruz, CA, USA) for 1 h and the reaction was visualized using the enhanced chemiluminescence (ECL) method (SuperSignal^®^West Pico Trial Kit, Rockford, IL, USA).

#### Enzyme-linked immunosorbent assays (ELISA)

The level of IL-1β in serum and CSF was determined using the rat IL-1β Platinum Kit (eBioscience, San Diego, CA, USA) according to the manufacturer’s instructions. The optical density of each well was determined at 450 nm using a Bio-Rad spectrophotometer (BioRad, Hercules, CA, USA).

#### Statistical analysis

Quantitative data are presented as means ± standard deviations (SD). Student’s t-test was used for comparison between two samples. The chi-square test was used to compare the difference between groups. Differences among more than two groups were assessed by one-way ANOVA with conservative Bonferroni’s test. Correlation analysis was achieved by Spearman rank correlation. All statistical analyses were performed on SAS for Windows 8.0 (SAS Institute Inc, Cary, NC, USA). Statistical significance was considered at a *p* < 0.05 level for all parameters.

## Additional Information

**How to cite this article**: Hu, C. *et al*. The role of inflammatory cytokines and ERK1/2 signaling in chronic prostatitis/chronic pelvic pain syndrome with related mental health disorders. *Sci. Rep.* **6**, 28608; doi: 10.1038/srep28608 (2016).

## Figures and Tables

**Table 1:** Clinical and mental parameters of control subjects and CP/CPPS patients.

	Controls	CP/CPPS
No. of subjects	992	810
Age (yrs.)	30.7 ± 8.40	32.2 ± 9.52
BMI (kg/m^2^)	23.1 ± 3.07	23.6 ± 3.41
NIH-CPSI	3.2 ± 5.03^*^	24.7 ± 6.39^*^
Anxiety (%)	17 (1.71%)^**^	247(30.50%)^**^
SAS	27.3 ± 4.98^*^	37.6 ± 7.51^*^
Depression (%)	99 (9.97%)^**^	351 (43.33%)^**^
SDS	32.1 ± 5.67^*^	40.4 ± 7.81^*^

CP/CPPS: chronic prostatitis/chronic pelvic pain syndrome; BMI: body mass index; NIH-CPSI: National Institutes of Health Chronic Prostatitis Symptom Index; SAS: self anxiety scale; SDS: self depression scale; Mean values ± SD are reported for all parameters. ^*^p < 0.0001 (Student’s t-test). ^**^p < 0.05 (chi-square test).

**Table 2:** Serum cytokine levels in control subjects and CP/CPPS patients, and their correlation with NIH-CPSI, SAS, and SDS.

Cytokines	Concentration (pg/ml)		Correlation coefficient (r)		
	Controls	CP/CPPS	NIH-CPSI	SAS	SDS
IL-1α	4.292 ± 0.435	8.617 ± 1.105*	0.144	0.020	−0.094
IL-1β	4.685 ± 0.727	7.840 ± 1.244*	0.232	0.438^**^	0.373^**^
IL-4	0.900 ± 0.119	3.287 ± 0.879*	0.020	0.005	−0.013
IL-6	4.867 ± 0.302	5.907 ± 0.733	0.009	−0.142^**^	−0.176^**^
IL-8	6.680 ± 0.79	8.015 ± 0.857	−2.586	−1.958	−2.366**
IL-10	0.866 ± 0.035	0.970 ± 0.057	−0.016**	−0.003	−0.005
IL-13	0.253 ± 0.017	0.579 ± 0.074*	−0.005	−0.002	−0.002
MCP-1	175.1 ± 5.873	191.5 ± 6.848	−1.449	0.514	−0.128
TNF-α	5.422 ± 0.486	8.151 ± 0.947*	0.425**	0.349**	0.290**
IFN-γ	2.091 ± 0.192	2.401 ± 0.282	0.007	−0.023	−0.057

IL: interleukin; MCP: monocyte chemokine protein; TNF: tumor necrosis factor; IFN: interferon; Mean values ± SD are reported for all parameters.

^*^CP/CPPS compared to controls when *p* < 0.05 (Student’s t-test).

^**^The index correlated to cytokines when *p* < 0.05 (Spearman rank correlation).

**Table 3:** The correlation of cytokine levels with behavioral performance in controls and the CP/CPPS rat model.

Cytokines	EPM	OF		Y maze	SBPA
	Time in open arms	Distance traveled	Ambulatory counts	Absolute latency	Latency
IL-1α	−0.161^**^	−0.000571	−0.000427	−0.0000814	−3.45*10^−7^
IL-1β	−0.339	−0.0167	−0.00583	−0.00476	−2.21*10^−6^
IL-4	−0.252	−0.000860	−0.000854	−0.000138	−6.85*10^−9^
IL-13	−0.000558	−0.0000180	−8.04*10^−6^	−7.78*10^−6^	−2.28*10^−8^
TNF-α	−0.270	−0.00137	−0.00104	−0.000462	−6.55*10^−8^

EPM: elevated plus maze; OF: open field; SBPA: shuttle box passive avoidance; ^**^The correlation coefficient correlating cytokines with behavioral performance when *p* < 0.05 (Spearman rank correlation).

**Table 4:** Primer sequences for cytokines and cytokine receptors.

Gene	Primer (From 5′ to 3′)	
IL-1α	Forward:	TGAGTCGGCAAAGAAATCAA
	Reverse:	GACAGATGGTCAATGGCAGA
IL-1β	Forward:	GCCAACAAGTGGTATTCTCCA
	Reverse:	CCGTCTTTCATCACACAGGA
IL-4	Forward:	ACCTTGCTGTCACCCTGTTC
	Reverse:	GTGTTCCTTGTTGCCGTAAG
IL-13	Forward:	CTCAGGGAGCTTATCGAGGA
	Reverse:	GCAACTGGAGATGTTGGTCA
TNF-α	Forward:	TGCCTCAGCCTCTTCTCATT
	Reverse:	GCTTGGTGGTTTGCTACGAC
IL-1R1	Forward:	CGCACGTCCTACACATACCA
	Reverse:	CATTCCGTGGGCTCATAATC
IL-1R2	Forward:	AAGTTGGTGTGGACGATGTTC
	Reverse:	GGGTGCTTCTCTGATGTAACG
IL-4R	Forward:	ACTGGCTGGAACTGTGGTCT
	Reverse:	TGGAGTGTGAGGTTGTCTGG
IL-13R1	Forward:	TTGATGACAACGACCTGTGG
	Reverse:	CACTGCGACAAAGACTGGAA
IL-13R2	Forward:	GGAATGCTGGGAAGGTTACA
	Reverse:	CAGTGTGGGTTCAGGGTCTT
TNFR1	Forward:	GAGGTGGAGGGTGAAGGAAT
	Reverse:	TGGAGACAGGATGACTGAAGC
TNFR2	Forward:	TTAGGACTGGCGAACTGCTT
GAPDH	Reverse:	GCCTTCCGTGTTCCTA
	Forward:	AGACAACCTGGTCCTCA
